# Supplementary material for: Improving the care of children with GENetic Rare disease: Observational Cohort study (GenROC)—a study protocol
Source: BMJ Open. 2024 May 16;14(5):e085237. doi: 10.1136/bmjopen-2024-085237 (PMC11103197; doi:10.1136/bmjopen-2024-085237)
Supplement: Supplementary data [file bmjopen-2024-085237supp001.pdf]

ACTB	CSNK2A1	ITPR1	NSD2	SMC1A
ACTL6B	CTCF	KANSL1	OPHN1	SMC3
ADNP	CTNNB1	KAT6A	PACS1	SON
AHDC1	CUL4B	KAT6B	PHIP	SOX5
ANKRD11	DDX3X	KCNQ2	PIGN	SPTAN1
ASH1L	DEAF1	KDM5B	POGZ	SRCAP
ASXL3	DPF2	KDM5C	PPP2R5D	STXBP1
ATP1A3	DYNC1H1	KIF1A	PRMT7	SYNGAP1
ATRX	DYRK1A	KMT2A	PUF60	TAF1
AUTS2	EBF3	KMT2C	PURA	TBL1XR1
BCL11A	EEF1A2	KMT5B	RAI1	TCF20
BPTF	EFTUD2	LZTR1	RERE	TCF4
BRPF1	ERF	MAGEL2	RPS6KA3	TLK2
BRWD3	FBX011	MECP2	SATB2	TRAPPC9
CACNA1A	FOXG1	MED12	SCN1A	TRIO
CAMTA1	FOXP1	MED13	SCN1B	TRIP12
CASK	GATAD2B	MED13L	SCN2A	TUBA1A
CDK13	GLI2	MEF2C	SCN8A	USP9X
CHD2	GRIK2	MYT1L	SETD1A	VPS13B
CHD3	GRIN1	NAA10	SETD5	WAC
CHD4	GRIN2A	NAA15	SHANK2	WDR45
CHD7	GRIN2B	NALCN	SHANK3	WDR62
CHD8	HECW2	NEXMIF	SIN3A	WDR73
CLTC	HNRNPU	NFIX	SLC6A1	ZBTB20
CNOT3	HUWE1	NRXN1	SLC6A8	ZMYND11
CNTNAP1	IQSEC2		SLC9A6	
