# Supplementary material for: Improving the care of children with GENetic Rare disease: Observational Cohort study (GenROC)—a study protocol
Source: BMJ Open. 2024 May 16;14(5):e085237. doi: 10.1136/bmjopen-2024-085237 (PMC11103197; doi:10.1136/bmjopen-2024-085237)
Supplement: Reviewer comments [file bmjopen-2024-085237.reviewer_comments.pdf]

PEER REVIEW HISTORY

BMJ Open publishes all reviews undertaken for accepted manuscripts. Reviewers are asked to complete a checklist review form (**http://bmjopen.bmj.com/site/about/resources/checklist.pdf**) and are provided with free text boxes to elaborate on their assessment. These free text comments are reproduced below.

ARTICLE DETAILS

**TITLE (PROVISIONAL)**	Improving the care of children with GENetic Rare disease: Observational Cohort study (GenROC): a study protocol
**AUTHORS**	Low, Karen; Watford, A; Blair, Peter; Nabney, I; Powell, John; Wynn, SL; Foreman, J; Firth, Helen; Ingram, Jenny

This article was not externally reviewed at BMJ Open. Protocols that have been independently assessed prior to submission to BMJ Open are usually fast-tracked to publication on the grounds that further substantial changes will not be possible. This independent assessment will usually be external, independent review for both a substantial grant award from a non-commercial or government funder and ethics approval.
